# Case Report: Complete response to the concurrent neoadjuvant radiation therapy and pembrolizumab in a locally recurrent, chemotherapy-refractory undifferentiated pleomorphic sarcoma of bone

**DOI:** 10.3389/fonc.2026.1645629

**Published:** 2026-02-12

**Authors:** Brina A. Patel, Jason T. Smith, Sintawat Wangsiricharoen, Wei-Lien Wang, Patrick P. Lin, Ahsan Farooqi, Elise F. Nassif Haddad, Kamal Ummed, Alexander F. Mericli, David M. Adelman, John Andrew Livingston, Anthony P. Conley

**Affiliations:** 1 The University of Texas at Austin College of Natural Sciences, Austin, TX, United States; 2 Department of Sarcoma Medical Oncology, The University of Texas MD Anderson Cancer Center, Houston, TX, United States; 3 Pathology and Laboratory Medicine, Oregon Health and Science University, Portland, OR, United States; 4 Department of Pathology, Division of Pathology/Lab Medicine, The University of Texas MD Anderson Cancer Center, Houston, TX, United States; 5 Department of Orthopedic Oncology, Division of Surgery The University of Texas MD Anderson Cancer Center, Houston, TX, United States; 6 Division of Radiation Oncology The University of Texas MD Anderson Cancer Center, Houston, TX, United States; 7 Georgia Cancer Specialists, Atlanta, GA, United States; 8 The University of Texas MD Anderson Cancer Center, Houston, TX, United States; 9 Department of Plastic Surgery The University of Texas MD Anderson Cancer Center, Houston, TX, United States

**Keywords:** bone sarcoma, chemotherapy, immunotherapy, sarcoma, undifferentiated pleomorphic sarcoma

## Abstract

Undifferentiated pleomorphic sarcoma of bone (UPS-B) is a rare and aggressive cancer that accounts for a small fraction of bone sarcomas. Compared to both undifferentiated pleomorphic sarcoma of soft tissue (UPS-ST) and osteosarcoma, UPS-B demonstrates lower chemosensitivity and a poorer prognosis, with reported five-year survival rates of only 7.3%. Standard management has generally mirrored osteosarcoma regimens, surgery, multi-agent chemotherapy, and radiotherapy, though the optimal approach remains undefined. In contrast, immune checkpoint inhibitors (ICIs) targeting PD-1/PD-L1 have shown promising activity in UPS-ST but minimal efficacy in osteosarcoma, leaving the role of ICIs in UPS-B largely unknown. We report the case of a 61-year-old male with recurrent, chemotherapy-refractory UPS-B of the left ilium who achieved a complete and durable response with concurrent neoadjuvant pembrolizumab and radiotherapy followed by surgical resection. The patient initially presented with a destructive iliac mass in 2021 and underwent induction with doxorubicin/cisplatin, which was complicated by acute kidney injury and thromboembolic events, followed by hemipelvectomy. He subsequently experienced local recurrence four months postoperatively, confirmed by biopsy. Treatment with high-dose ifosfamide provided no durable disease control, and imaging demonstrated progressive growth. Molecular profiling revealed 90% PD-L1 expression, multiple oncogenic mutations including SMARCA4 and POLE, and a mutational signature consistent with high tumor mutational burden (TMB), suggesting potential immunogenicity. Based on these features and disease progression, he was treated with hypofractionated radiotherapy (42.75 Gy in 15 fractions) concurrently with pembrolizumab for three cycles prior to surgery. Resection in June 2022 demonstrated no viable tumor, consistent with complete pathologic response. The patient has since completed one year of adjuvant pembrolizumab, with ongoing therapy and no evidence of recurrence at 24 month follow-up.

## Introduction

Undifferentiated pleomorphic sarcomas (UPS) are a highly aggressive type of cancer that can develop in either soft tissues or bones, but UPS of bone (UPS-B) is exceedingly rarer and has a worse prognosis than UPS of the soft tissue (UPS-S) (1, 2). The distinction between UPS-S and UPS-B is significant, as it highlights the need for treatment tailored to the specific type of UPS. Traditionally, UPS-B has been treated similarly to primary bone tumors like osteosarcoma, using surgery and combination chemotherapy regimens containing methotrexate, cisplatin, and doxorubicin (1, 3), but the optimal chemotherapy course for UPS-B remains unclear (3, 4). It is important to note that studies have also highlighted UPS-B’s lower chemosensitivity than osteosarcoma (3, 5, 6). The 5-year survival rate for UPS-B patients is around 7.3%, considerably lower than that of osteosarcoma at 77% (1, 2, 5).

**Figure 1:**
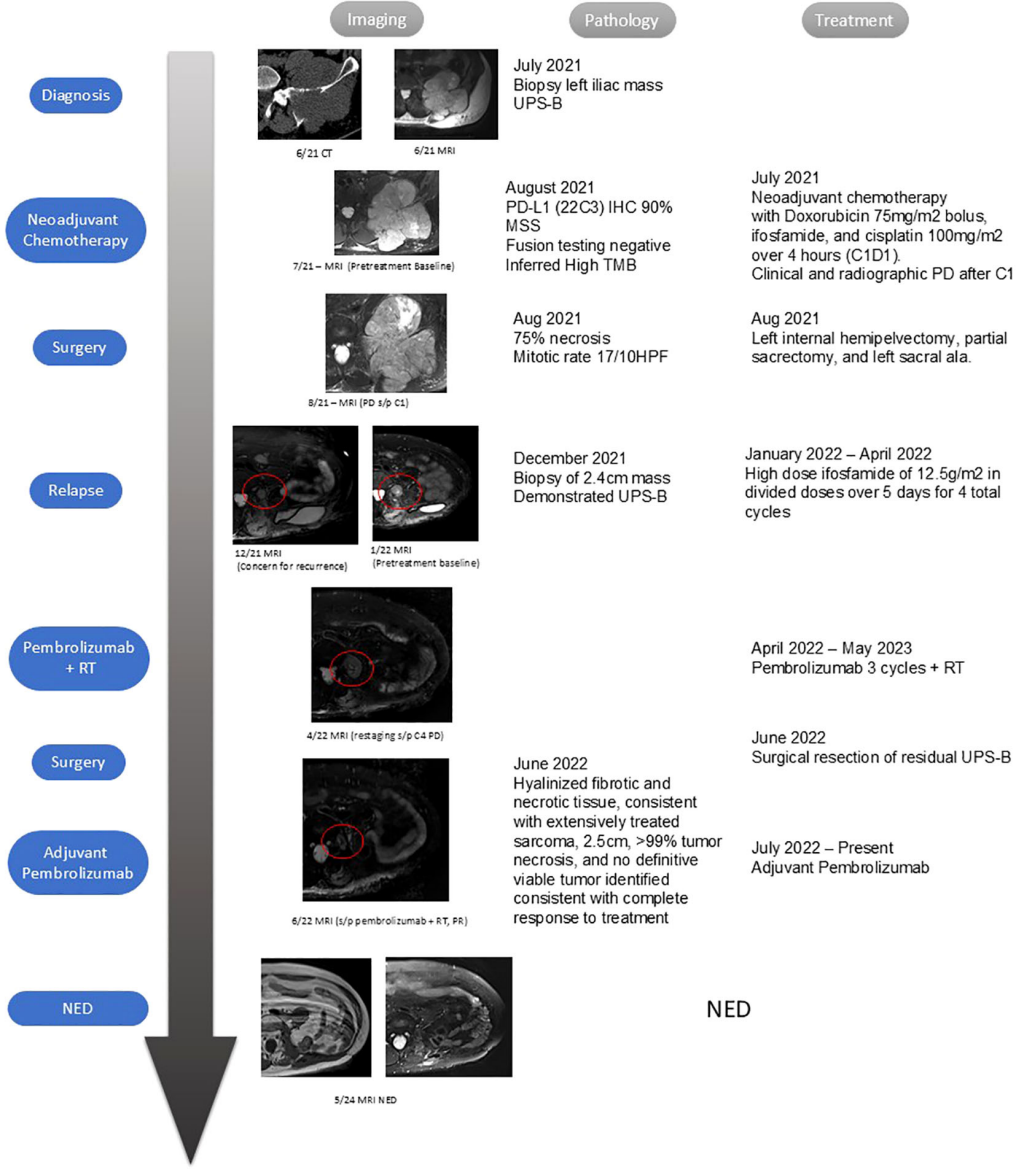
Chronological progression of patient exams and treatments. Stage of treatment is listed from top to bottom, with dates of related imaging, pathology findings, and subsequent treatments from left to right.

Immune checkpoint inhibitors (ICIs), which target the PD-1/PD-L1 pathway to boost immune response against cancerous cells, have revolutionized cancer care across different cancer types but unfortunately have very limited efficacy in osteosarcomas (7, 8). However, ICIs have been shown to be effective in advanced UPS-S: in a study examining the safety and efficacy of pembrolizumab in advanced sarcomas, UPS-S emerged as being particularly sensitive to immunotherapy, with a 23% objective response rate, suggesting the potential of this class of drugs for this sarcoma subtype (9, 10). Two studies evaluated neoadjuvant ICI for surgically resectable UPS-S in combination with radiation therapy (RT): the first was a phase 2 randomized trial with nivolumab (PD-1 inhibitor) ± ipilimumab (CTLA4 inhibitor), which demonstrated a pathologic response rate of 89% in 10 patients with UPS-S, and the second trial, SARC032, was a randomized trial investigating the addition of pembrolizumab to standard of care (SOC) RT in stage 3 soft-tissue sarcoma (85% of patients had UPS-S) and demonstrated a significant improvement in 2-year disease-free survival from 53% to 70% (11). Collectively, these findings suggest that concurrent ICI and RT may be more effective than single-agent therapy for treating UPS-S, but the response of UPS-B to this strategy remains largely unclear. Notably, it is unknown whether UPS-B will have a similar response to ICI as osteosarcoma or as UPS-S. Herein, we report on a case of a patient with locally recurrent ups-B with a complete durable response to a combination of pre-operative ICI and RT (**Figure 1**).

## Case description

**Figure 2:**
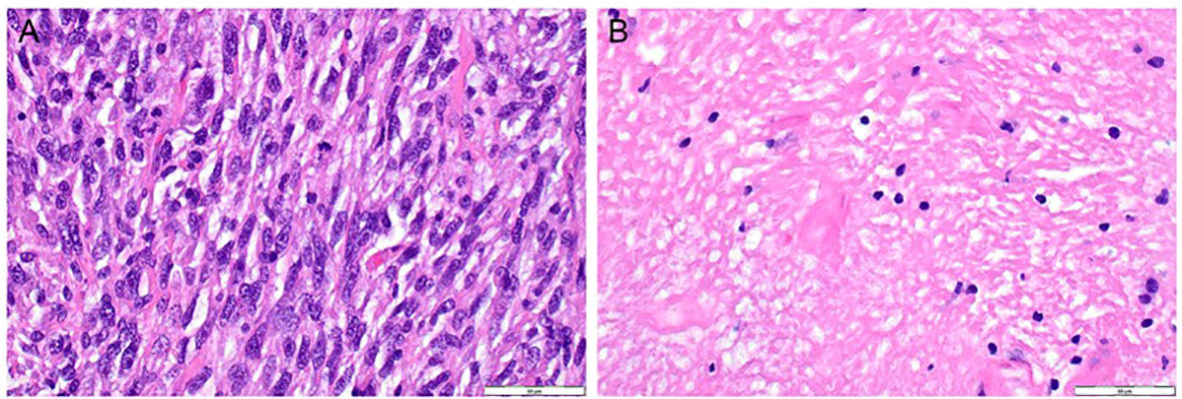
Representative Pathology Slides Before and After Treatment. **(A)** Pre-treatment, December 2021, H&E, 400x. **(B)** Post-treatment, June 2022, H&E, 400x. Extensive hyalinization with scattered lymphocytes.

A 61-year-old male with a past medical history of osteoarthritis, benign prostatic hyperplasia, repaired hiatal hernia, hypertension, neuropathy, and a stable pulmonary nodule for 10 years presented with progressive left hip pain in February 2021, soon noticing a palpable left hip mass in June 2021. Imaging of the mass conducted in June 2021 displayed a large destructive tumor arising in the left upper iliac bones with cortical breakthrough of soft tissue to the left psoas muscle (11.0 cm × 12.0 cm × 10 cm). In July 2021, a biopsy of the tumor was performed and revealed a UPS-B with an observed mitotic rate of 7 per single high-power field and the presence of foci of necrosis (**Figure 2A**).

**Figure 3:**
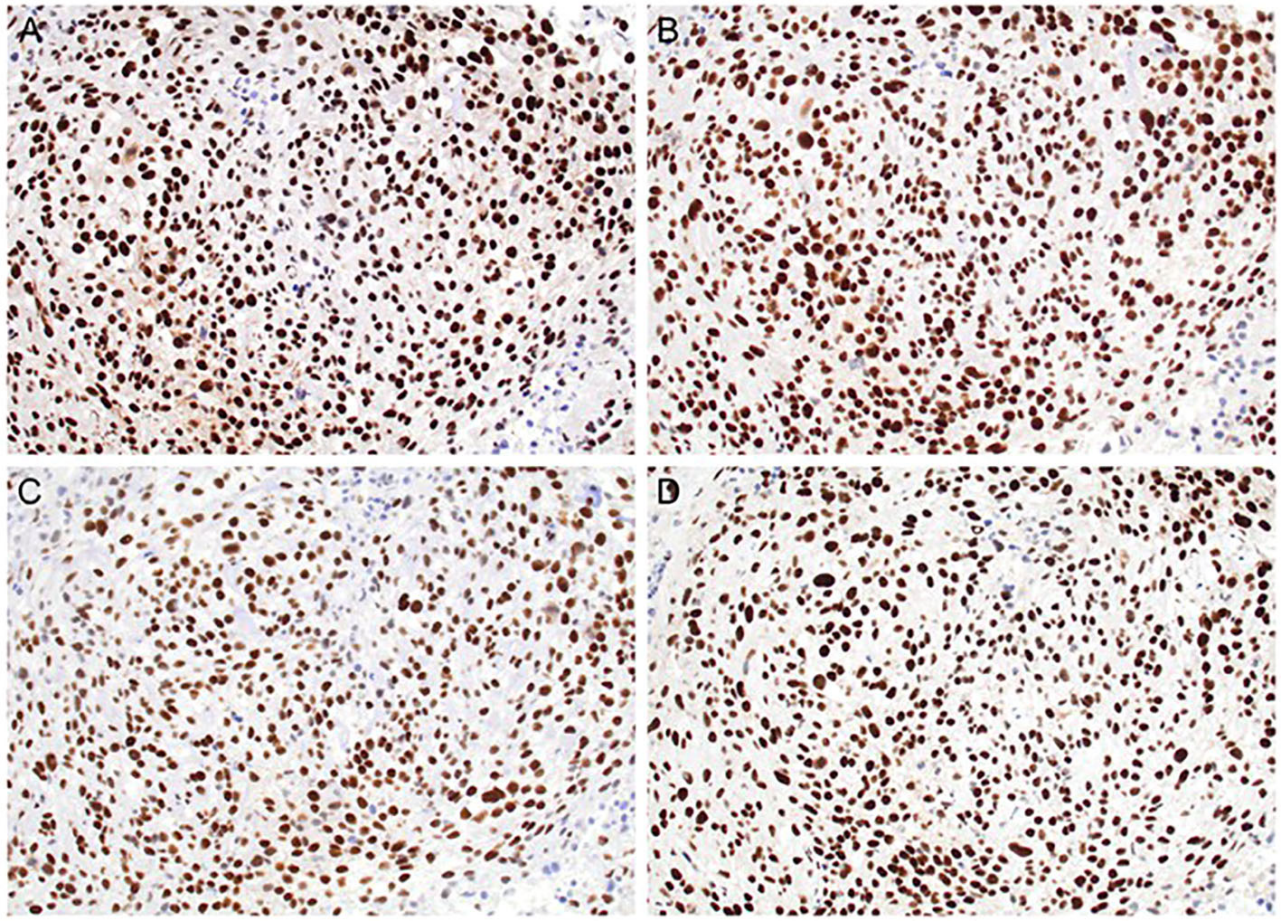
Mismatch repair (MMR) protein immunohistochemistry demonstrating retained nuclear expression of all four markers at 200× magnification. **(A)** MLH1, **(B)** MSH2, **(C)** PMS2, and **(D)** MSH6. Retained expression across all markers is consistent with proficient mismatch repair status.

A Solid Tumor Genomic Assay was conducted using next-generation sequencing (NGS) through a targeted 500-gene panel (FoundationOne CDx). Variant calling was performed using GRCh37/hg19 as the reference genome. Variants were prioritized based on allele frequency, predicted pathogenicity, cancer-relevance annotation, and presence in oncogenic pathways. Copy-number alterations were assessed using NGS-based computational CNV algorithms as part of the FoundationOne CDx analytic pipeline. On the biopsy specimen, the resulting DNA report highlighted multiple missense mutations in oncogenes and tumor suppressor genes: ARID1A, ATR, AXL, BRAF, CDKN2A, CHEK1, ERBB2, EZH2, FANCA, FBXW7, FGFR2, NBN, NF1, NTRK3, POLE, RAC1, ROS1, SMARCA4, STK11, TERT, TP53, and TSC1. Germline DNA was not available for comparison; therefore, the identified alterations are described as tumor-identified variants rather than definitively somatic. The presence of numerous tumor-identified alterations across oncogenic pathways suggests a relatively elevated mutational burden based on this targeted panel, which may be associated with increased tumor immunogenicity (**Table 1**). Immunochemistry demonstrated retained nuclear expression of the mismatch repair proteins MLH1, MSH2, MSH6, and PMS2, consistent with proficient mismatch repair (**Figure 3**). PD-L1 immunohistochemistry using clone 22C3 showed a tumor proportion score of 90%, with membranous staining in 90% of viable tumor cells. NY-ESO-1 immunohistochemistry was negative, with no tumor cell labeling observed (0%) (data not shown).

**Table 1:** Solid tumor genomic assay.

Somatic mutations					
Gene	Standardized nomenclature (HGVS)	Location	DNA change	Variant type	COSMIC ID
ARID1A	NM_006015.6(ARID1A):c.1991C>T p.S664L	Exon 5	SNV	Missense	
ARID1A	NM_006015.6(ARID1A):c.3932C>Tp.P1311L	Exon 16	SNV	Missense	
ATR	NM_001184.4(ATR):c.7597C>T p.R2533*	Exon 45	SNV	Nonsense	COSM35421
AXL	NM_001699.6(AXL):c.1778-1G>A p.?	Splice (intron 14)	Splice	Unknown	
BRAF	NM_004333.6(BRAF):c.1790T>G p.L597R	Exon 15 18	SNV	Missense	COSM471
CDKN2A	NM_000077.4(CDKN2A):c.341C>T p.P114L	Exon 2	SNV	Missense	COSM12476
CHEK1	NM_001274.5(CHEK1):c.466C>T p.R156W	Exon 6	SNV	Missense	
ERBB2	NM_004448.3(ERBB2):c.2690_2691delinsAA p.R897Q	Exon 22	Complex	Missense	
EZH2	NM_004456.5(EZH2):c.2068C>T p.R690C	Exon 18	SNV	Missense	COSM49154
FANCA	NM_000135.4(FANCA):c.2695C>T p.P899S	Exon 28	SNV	Missense	
FBXW7	NM_033632.3(FBXW7):c.379C>T p.Q127*	Exon 2	SNV	Missense	
FGFR2	NM_000141.4(FGFR2):c.1605G>A p.M535I	Exon 12	SNV	Missense	
FGFR2	NM_000141.4(FGFR2):c.1115C>T p.S372F	Exon 9	SNV	Missense	
FGFR2	NM_000141.4(FGFR2):c.961G>A p.D321N	Exon 8	SNV	Missense	
FGFR2	NM_000141.4(FGFR2):c.838G>A p.V280l	Exon 7	SNV	Missense	
NBN	NM_002485.4(NBN):c.1190C>T p.S397L	Exon 10	SNV	Missense	
NF1	NM_001042492.3(NF1):c.4006C>T p.Q1336*	Exon 30	SNV	Nonsense	COSM28019
NTRK3	NM_002530.4(NTRK3):c.2426T>A p.l809N	Exon 19	SNV	Missense	
POLE	NM_006231.4(POLE):c.4513C>T p.P1505S	Exon 35	SNV	Missense	
RAC1	NM_006908.5(RAC1):c.86C>T p.P29L	Exon 2	SNV	Missense	
ROS1	NM_002944.2(ROS1):c.5885G>A p.G1962E	Exon 36	SNV	Missense	
SMARCA4	NM_003072.4(SMARCA4):c.415C>T p.P139S	Exon 4	SNV	Missense	
SMARCA4	NM_003072.4(SMARCA4):c.2438C>T p.?	Splice? (Exon 16)	SNV	Unknown	
STK11	NM_000455.5(STK11):c.824C>G p.P275R	Exon 6	SNV	Missense	
TERT	NM_198253.3(TERT):c.-124C>T	UTR5	SNV		
TP53	NM_000546.5(TP53):c.722C>T p.S241F	Exon 7	SNV	Missense	COSM10812
TSC1	NM_000368.5(TSC1):c.2047C>T p.P683S	Exon 17	SNV	Missense	

Summary of variants identified in the patient's solid tumor genomic assay. Notable genes with detected variants include ARID1A, ATR, AXL, BRAF, CDKN2A, CHEK1, ERBB2, EZH2, FANCA, FBXW7, FGFR2, NBN, NF1, NTRK3, POLE, RAC1, ROS1, SMARCA4, STK11, TERT, TP53, and TSC1. Genomic locations are provided for each variant, and coordinates are reported according to the GRCh37 (hg19) human genome assembly.

The multidisciplinary recommendation was for the patient to receive neoadjuvant chemotherapy following osteosarcoma-like regimens. The patient began a course of doxorubicin 75 mg/m^2^ bolus, zinecard, and cisplatin 100mg/m^2^ over 4h in the inpatient setting in July 2021 (C1D1). However, the initial treatment plan was complicated by acute kidney injury, with an increase in creatinine from 0.91 mg/dl to 2.91 mg/dl. In August 2021, the patient developed multiple thrombosis emboli from the right subclavian vein and pulmonary embolism. Post C1, the patient reported an enlargement of the mass, which was confirmed with an MRI in August 2021, revealing the mass measured 15.5 cm × 14.3 cm × 12 cm. Thus, the chemotherapy was discontinued in August 2021, and the patient underwent surgical resection consisting of a left internal hemipelvectomy, partial sacrectomy, and left sacral ala in August 2021. Postoperative complications included upper extremity weakness (transient ischemic attack) and surgical site infection requiring debridement and washout of the left pelvic and abdominal flank. Additionally, the patient was managed for an infection at the surgical site and underwent debridement and washout of the left pelvic and abdominal flank by long-term antibiotic therapy to address MSSA bacteremia as a source of pelvic abscess.

In December 2021, 4 months after the surgery, an MRI of the pelvis noted a soft tissue process measuring 1.8 cm × 1.6 cm along the medial margin of the resection site, adjacent to the superolateral osteotomy of the right sacral ala, concerning for recurrent disease (**Figure 1** labeled as UPS-B relapse). A biopsy was performed in December 2021 and demonstrated recurrent UPS. Restaging imaging in January 2022 noted an interval increase in the size of the tumor, now measuring 2.4 cm × 2 cm. From January 2022 to February 2022, the patient was treated with a high dose of ifosfamide of 12.5 g/m^2^ in divided doses over 5 days, consistent with osteosarcoma-like treatment. After his second cycle, an imaging evaluation with MRI showed a stable tumor size, leading the patient’s course of treatment to be continued. However, the patient’s imaging after the fourth cycle demonstrated an increase in size of 3.4 cm (previously 2.7 cm). This course of therapy was discontinued due to progressive disease in April 2022, and it was recommended the patient undergo preoperative RT followed by surgery.

Based on the patient’s favorable molecular biology (high TMB, PD-L1 of 90%, and mutations in SMARCA4 and POLE genes), previous progression through systemic chemotherapy, based on SARC028 data demonstrating activity of pembrolizumab for UPS-S, and previous experience demonstrating activity of nivolumab and ipilimumab in conjunction with RT for UPS-S, he was recommended to proceed with RT with concurrent pembrolizumab therapy for three cycles between April and May 2022. The patient tolerated this course of treatment well without any toxicities.

In June 2022, the patient underwent his final surgical resection. Final pathology noted hyalinized fibrotic and necrotic tissue, consistent with extensively treated sarcoma, 2.5 cm, >99% tumor necrosis, and no definitive viable tumor identified, consistent with complete response to treatment (**Figure 2B**). The patient continued adjuvant pembrolizumab to complete 1 year of therapy. During that time in January of 2023, the patient did develop a mild rash on the left upper back, which was treated with topical steroid cream (Grade 1). In May of 2023, the patient started post-operative pembrolizumab. The patient remains on pembrolizumab treatment without evidence of disease as of the last follow-up in May 2024.

## Discussion

The exceptional response of this patient poses a question in the approach of the typical treatment of UPS-B: should we begin to consider UPS-B as an immune-sensitive type of tumor like UPS-S?

There is a possibility that this great pathologic response may be due to the combination of ICI and RT, as a previous study examining the use of neoadjuvant ICI and RT therapy in patients with UPS and DDLPS has shown a much higher median pathologic response in UPS-S (89%) compared to DDLPS patients (22.5%) with improved disease-free survival (12, 13). Recently, the SARC032 confirmed the synergy of RT and ICI in patients with localized UPS-S, showing a clear benefit in disease-free survival (14). However, despite good responses noted in patients with UPS-S, some patients with UPS-S are resistant to ICI, and reliable biomarkers of response are lacking. In this case, the patient received RT with concurrent pembrolizumab therapy due to the histologic type (UPS) and after failure of other osteosarcoma-like SOC chemotherapies.

Specifically, the tumor harbored oncogenic mutations in SMARCA4, POLE, and STK11, which have been associated with response to ICIs (15). Additionally, this tumor had a high number of mutations on the limited NGS targeted panel, suggesting a high TMB, which has been associated with response to ICI across cancer types (16). Yet, biomarkers of response to ICIs are not well defined in sarcomas, and there is a general uncertainty in patient selection since there are no clear indicators of who will respond well to ICI, leading to limited treatment efficacy.

Although osteosarcomas exhibit an inflamed tumor microenvironment, they paradoxically respond poorly to ICI, suggesting differential immune sensitivity among bone sarcoma subtypes and emphasizing the need for further research to understand these disparities (17). Biomarkers will be crucial to target and overcome low immunogenicity and therapeutic resistance in sarcomas (18). In sarcomas, recent research suggests that the presence of tertiary lymphoid structures (TLS) in a tumor is associated with better response to ICI treatment (19, 20). In a phase 2 trial examining pembrolizumab combined with low-dose cyclophosphamide for advanced sarcomas (PEMBROSARC trial), the trial’s primary endpoint of a 6-month non-progression rate (NPR) was achieved after specifically selecting patients with TLS (40% NPR) with an overall improved objective response rate (30% from 2.4%) and a median progression-free survival of 4.1 months (20). These findings may relate to the current case by suggesting that UPS-B tumors with TLS or a highly inflamed phenotype could demonstrate improved ICI responsiveness.

Limitations of this study include its single-patient nature and the lack of direct TLS or immune infiltration assessment. Future studies should include immune profiling and multi-omic analyses of UPS-B to identify predictive biomarkers of ICI response. Additionally, prospective studies evaluating the RT and ICI combination in UPS-B are warranted.

## Data Availability

The original contributions presented in the study are included in the article/supplementary material. Further inquiries can be directed to the corresponding author.
